# Transcriptomes of Zebrafish in Early Stages of Multiple Viral Invasions Reveal the Role of Sterols in Innate Immune Switch-On

**DOI:** 10.3390/ijms24054427

**Published:** 2023-02-23

**Authors:** Gang Ouyang, Le Yuan, Xiao-Qin Xia, Wanting Zhang, Mijuan Shi

**Affiliations:** 1 State Key Laboratory of Freshwater Ecology and Biotechnology, Institute of Hydrobiology, Chinese Academy of Sciences, Wuhan 430072, China; 2 The Key Laboratory of Aquaculture Disease Control, Ministry of Agriculture, Wuhan 430072, China; 3 The Innovation of Seed Design, Chinese Academy of Sciences, Wuhan 430072, China; 4 Hubei Hongshan Laboratory, Wuhan 430070, China; 5 University of Chinese Academy of Sciences, Beijing 100049, China

**Keywords:** early stage of viral infection, stress, steroids, switch-on innate immune

## Abstract

Although it is widely accepted that in the early stages of virus infection, fish pattern recognition receptors are the first to identify viruses and initiate innate immune responses, this process has never been thoroughly investigated. In this study, we infected larval zebrafish with four different viruses and analyzed whole-fish expression profiles from five groups of fish, including controls, at 10 h after infection. At this early stage of virus infection, 60.28% of the differentially expressed genes displayed the same expression pattern across all viruses, with the majority of immune-related genes downregulated and genes associated with protein synthesis and sterol synthesis upregulated. Furthermore, these protein synthesis- and sterol synthesis-related genes were strongly positively correlated in the expression pattern of the rare key upregulated immune genes, IRF3 and IRF7, which were not positively correlated with any known pattern recognition receptor gene. We hypothesize that viral infection triggered a large amount of protein synthesis that stressed the endoplasmic reticulum and the organism responded to this stress by suppressing the body’s immune system while also mediating an increase in steroids. The increase in sterols then participates the activation of IRF3 and IRF7 and triggers the fish’s innate immunological response to the virus infection.

## 1. Introduction

The primary and important line of defense against viral invasion in fish is the innate immune system, which is made up of cellular, humoral, and physical barriers [1]. As the sentinels in innate immunity, the pattern recognition receptor (PRR) genes have attracted the most attention in immunology research, which has been heavily focused on changes in major innate immunity-related genes in fish triggered by a particular viral infection through PRRs [2,3,4,5]. The detection of the non-self is the first and most important stage in the induction of the innate immune response. However, in virus-infected fish, does the PRR actually represent the first stage of the immune response? Is there another system that controls this procedure? If so, does this system recognize viruses differently than PRR? All of these issues have yet to be researched.

For example, the immune system in fish can be controlled by the neuroendocrine system [6,7]. Numerous studies have shown that the neuroendocrine system regulates the synthesis of specific substances, such as hormones, in the body under stressful conditions, influencing fish immunity [8,9,10]. This modulation depends on the stress time course and is multifaceted, not just immune-suppressive or immune-enhancing [11,12]. Research on the substances involved in this intricate regulatory process is likewise scarce, concentrating only on catecholamines and a few other sterol hormones, such as adreno-corticotropic hormone, corticosteroid-releasing hormone, and glucocorticoids [11,12,13]. The hypothalamus–pituitary–gonadal axis and the hypothalamus–pituitary–interrenal axis regulate steroid production in teleost fish. According to Tokarz et al. (2015) [14], all genes associated with steroidogenesis have been found in different species, demonstrating the significance and universality of these tiny molecules. These substances are engaged in numerous critical fish life processes.

In reality, in addition to steroid hormones, different types of cholesterol have a role in viral resistance as non-hormonal steroids. For instance, 25-hydroxycholesterol (25HC) can increase the body’s viral resistance by suppressing the fusion of numerous viruses’ membranes with host cells. Cholesterol 25-hydroxylase, which converts cholesterol to 25HC, is also one of the interferon (IFN) target genes [15,16]. Furthermore, 7-dehydrocholesterol (7-DHC) can increase IFN-I production by promoting the phosphorylation of interferon regulatory factor 3 (IRF3) [17]. This study suggests that sterols may play a role in activating the innate system.

In our study, zebrafish at 3 days post-fertilization (dpf) were chosen as samples [18] to eliminate the effects of acquired immunity. They were infected with four viruses including two DNA viruses (crucian carp herpesvirus (CAHV) [19] and Cyprinid Herpesvirus 2 (CyHV2) [20]) and two RNA viruses (spring viraemia of carp virus (SVCV) [21] and grass carp reovirus II (GCRV II) [22]). Most of the important freshwater fish farmed in China are cyprinids, such as grass carp, common carp, and crucian carp. These four viruses are the pathogenic viruses of the major viral diseases of these economically-important fish. The expression profile of fish infected with each virus for 10 h revealed that at the start of viral infection, a generalized system may exist in fish to turn on innate immunity. This system recognizes the virus before PRRs and may be activated by excessive protein synthesis. This mechanism then promotes the synthesis of sterol molecules to mediate the overexpression of IRF3 and IRF7 [23] and thus provide protection against viruses.

## 2. Results

### 2.1. Transcriptome Assembly and Statistics

Four viruses were used to immerse zebrafish larvae, which lack the acquired immune system, in order to study the first immune response of fish to infections by various viruses. In total, 15 zebrafish samples consisting of 3 replicates from each of the 5 zebrafish groups (4 treatments and a control) were subjected to RNA-seq.

The mapping rate is 93.27 ± 0.11% and the clean data are 43.59 ± 6.24 M reads. The data were reduced to 32.41 ± 4.28 M reads after clustering by unique molecular identifiers (UMIs). Evidently, the standard deviation of readings for each sample was also lowered (Table S1).

Reads were assembled into a transcriptome of 107,348 transcripts from 62,671 genes using NCBI’s most recent GFF file (GRCz11). In total, 26,370 of these genes only encode ncRNA (non-coding RNA), while 4146 mRNA-coding genes were transcribed at a very low level. The count matrix of the remaining 32,155 genes was used for differential expression analysis after these genes were removed.

### 2.2. Preliminary Statistics of Differential Expression Genes (DEGs)

Two comparisons were done between the gene expression levels of the five groups: (1) comparisons between each of the four viral treatment groups and the control; and (2) comparisons between the treatments of two DNA viruses (CaHV vs. CyHV2) and two RNA viruses (GCRV II vs. SVCV). Comparisons with a 0.05 level of significance yielded DEGs.

**Figure 1:**
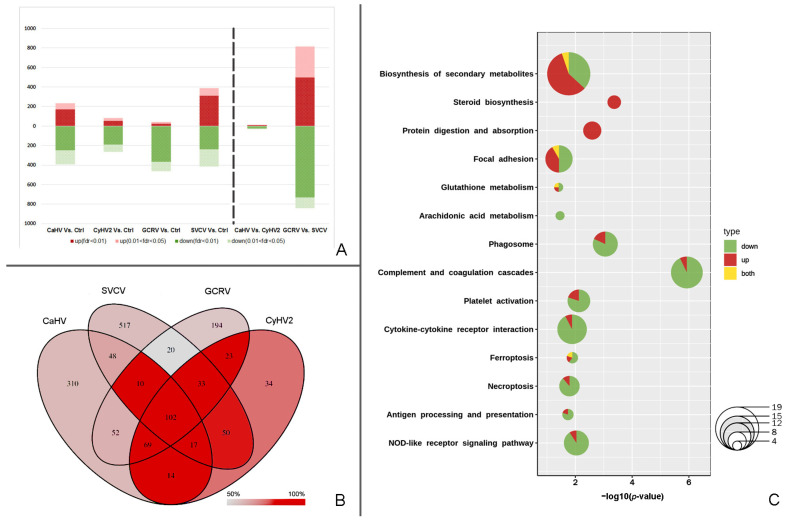
Summary diagrams of DEGs and PBGs. (**A**) Stacked bars of DEGs. (**B**) The Venn diagram of DEGs in the infections by four viruses. The depth of red is in accord with the proportion of PBGs. (**C**) Bubble pie chart of enriched pathways based on PBGs. Red indicates upregulated PBGs, green indicates downregulated PBGs, and yellow indicates genes with multiple copies, some of which are upregulated and the others downregulated.

CyHV2 produced the fewest DEGs of the four viruses, while SVCV produced the most. The GCRV II-induced DEGs were highly imbalanced, with up to 91.88% being downregulated. Furthermore, there are only 45 DEGs between the treatments of two DNA viruses, whereas there are 1656 between the treatments of two RNA viruses (Figure 1A).

### 2.3. Potential Basic Genes and Pathway Enrichment

In the four treatments, 1493 genes appear as DEGs at least once (Figure 1B). In all four treatments, the expression of 900 genes (60.28%) among these DEGs showed the same expression trend vs. control. In this study, these 900 genes were identified as potential basic genes (PBGs). The proportion of PBGs in each Venn diagram region of Figure 1B ranges from 50% to 100%. The PBGs are commonly found in overlapping regions of multiple treatments. For example, there are 102 genes that are differentially expressed in all four treatments, and all of them are PBGs. Based on the 900 PBGs, fourteen pathways were enriched (Figure 1C). These pathways are roughly divided into two groups: metabolism and immunity. The upregulated PBGs were mostly found in metabolic pathways. All PBGs were upregulated, particularly in the “steroid biosynthesis” and “protein digestion and absorption” pathways. A higher proportion of downregulated PBGs were found in immune-related pathways, with the complement and coagulation cascades pathway having the most distinct enrichment.

### 2.4. Horizontal Comparison of Pathway Enrichment in the Initial Stage of Infections

**Figure 2:**
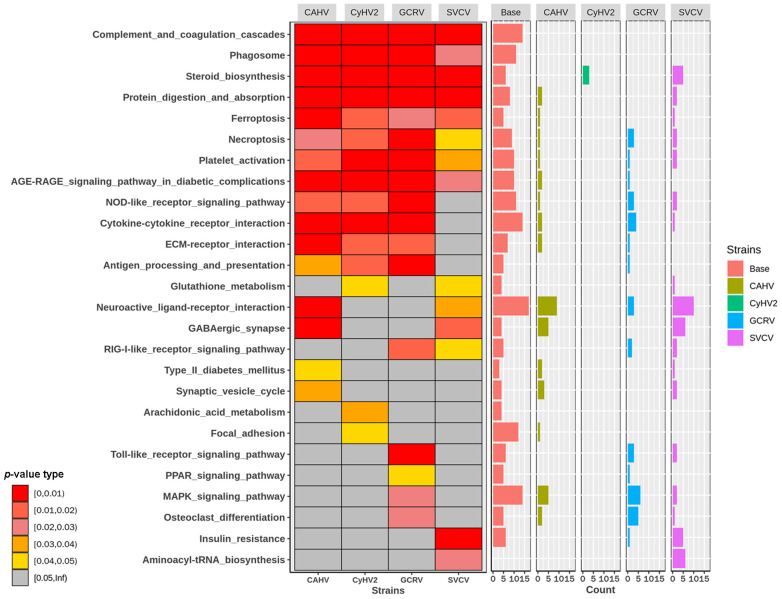
Diagrams of enriched pathways based on DEGs and PBGs in infections by four viruses, respectively. Heat map: The *p*-values of the enriched pathway based on the DEGs and PBGs per virus. Bar plot: The number of PBGs and non-PBGs per virus in each enriched pathway.

For the pathway-enriched analysis, both PBGs and other DEGs (non-PBGs) for each virus were used to study the impacts. For each virus, the *p*-value and the number of PBGs and non-PBGs in each enriched pathway were displayed separately (Figure 2). CyHV2 induces very few non-PBGs, which are only enriched in the steroid biosynthesis pathway. The SVCV induced most non-PBGs, and notably, the aminoacyl–tRNA biosynthesis pathway only has DEGs during SVCV infection.

**Figure 3:**
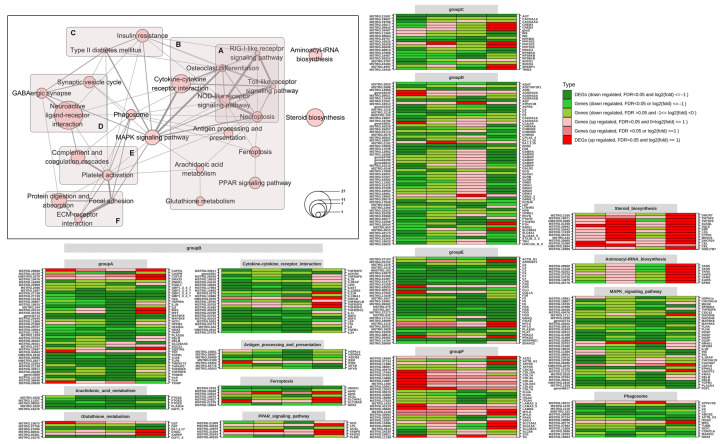
The network of enriched pathways and heat maps of DEGs. The order of four viruses in all the heat maps is CaHV, CyHV2, GCRV, and SVCV.

Then, the number of common genes and pathway-specific genes was used to gauge the link between these enriched pathways because many genes are shared across multiple pathways. According to their biological function, the pathways are divided into six groups (groups A through F) (Figure 3).

Group A, a subset of group B, is the main set of immune system pathways. It consists of five pathways that have several genes in common. With the exception of three DEGs in CaHV infection and six in SVCV infection, DEGs in group A were generally downregulated (Figure 3). Group B was an immune-related pathway set that included group A and six other pathways (arachidonic acid metabolism, glutathione metabolism, antigen processing and presentation, cytokine–cytokine receptor interaction, ferroptosis, and PPAR signaling pathway). Other DEGs in group B, like those in group A, were mostly downregulated during viral infection. The DEGs of the arachidonic acid metabolism pathway, in particular, were all downregulated without regard for virus specificity. Except for the antigen processing and presentation pathway, most of the upregulated genes in group B were enriched in SVCV infection.

The upregulated genes in group C, which consists of the two insulin-signaling pathways, were also enriched in SVCV infection. Group D is a collection of pathways involved in the transmission of nerve signals. CaHV, CyHV2, and SVCV infections downregulated most of the genes in this group to varying degrees, while GCRV II infection slightly upregulated most of the same genes. In the initial phase of viral infection, the majority of platelet and complement pathway genes (group E) were downregulated. On the other hand, after infection with various viruses, the genes in intercellular communication pathways (group F) were mostly upregulated at various levels. Genes of the collagen family, in particular, were upregulated in all viral infections and even expressed significantly differently in CaHV infection. The MAPK signaling pathway and the phagosome pathway are the hub pathways in the pathway network, and they share genes with many other pathways. The majority of genes in these two pathways were downregulated. Steroid biosynthesis and aminoacyl–tRNA biosynthesis, on the other hand, share no genes with any other pathway enriched in this study. The number of upregulated DEGs in the steroid biosynthesis pathway in all treatments was ranked as SVCV > CyHV2 > CaHV > GCRV II, where no DEG was detected in GCRV II infection. Genes in the aminoacyl–tRNA biosynthesis pathway were all significantly upregulated in SVCV infection, but not in the other three viral infections.

### 2.5. Differential Expressive Analysis under Different Classification Levels of Viruses

**Figure 4:**
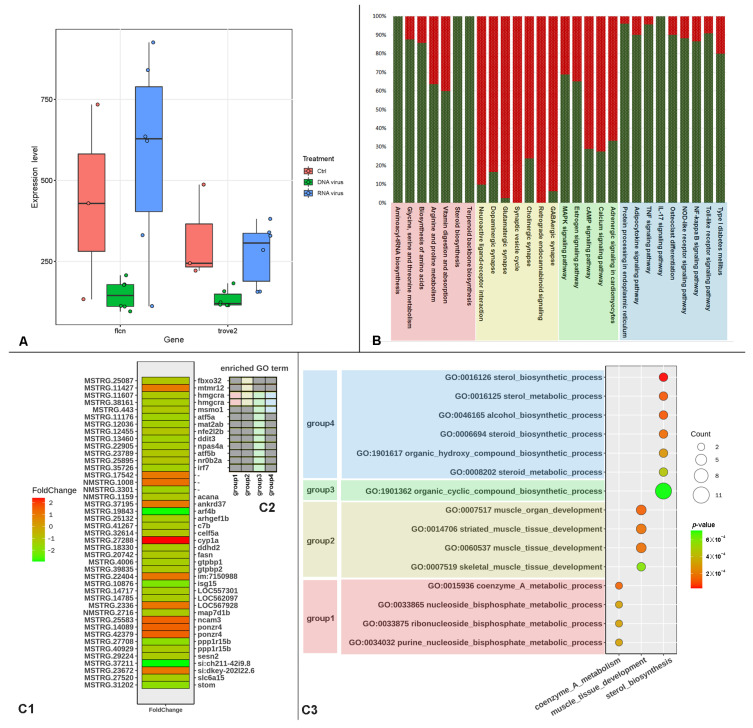
Summary of DEG analysis by dimensions. (**A**) Expression levels of two DEGs between DNA virus and RNA virus. (**B**) Percentage stacked bar chart of the enriched pathways based on DEGs between infections by two RNA viruses. Red block: metabolic-related pathways. Yellow block: nerve signal transmission pathways. Green block: signal transduction pathways. Blue block: immune-related pathways. (**C**) DEGs and enriched GO terms between the infections by two DNA viruses. (**C1**) The heat map of the log2 (fold change) of 43 DEGs with nr alignments. (**C2**) Grid graph to show DEGs corresponding to the four groups of enriched GO terms. (**C3**) The first 15 enriched GO terms of biological process (BP) with the lowest *p*-value. The x-axis is for the function categories of the GO terms. The y-axis coordinate was the four groups of GO terms.

First, only two DEGs were identified following a comparison of DNA viruses (CaHV and CyHV2) and RNA viruses (GCRV II and SVCV). The expression levels of these two genes remained relatively constant across individuals during DNA virus infections but fluctuated more in control and RNA virus infections (Figure 4A).

Second, the profiles of two RNA viruses, GCRV II (dsRNA) vs. SVCV (ssRNA), differ significantly, with a total of 1656 DEGs, including 815 upregulated DEGs and 841 downregulated DEGs. In total, 28 pathways were enriched based on these DEGs (*p* ≤ 0.05, Figure 4B). The enriched pathways were classified into four categories based on their functions: immune-related pathways, signal transduction pathways, nerve signal transmission pathways, and metabolic-related pathways. Across these categories, the proportions of upregulated and downregulated genes change. Downregulated DEGs are more prevalent in metabolic- and immune-related pathways than upregulated DEGs. The proportions of upregulated and downregulated DEGs in signal transduction pathways are not significantly different. However, in the neural signaling pathways, upregulated DEGs are significantly more common than downregulated DEGs.

Third, only 45 DEGs were found between the infections caused by the two DNA viruses (CaHV and CyHV2), and two of these have no homologs in the nr database. Eleven of the remaining DEGs were upregulated, while 32 were downregulated (Figure 4 (C1)). The first 15 GO terms with the lowest *p*-value in biological process (BP) enrichment were classified into three groups: sterol biosynthesis, muscle tissue development, and coenzyme A metabolism (Figure 4 (C3)). In total, 13 DEGs are linked to the top 15 enriched GO terms. Additionally, the number of genes associated with various GO terms within a single category is typically the same. However, the organic cyclic compound biosynthetic process (GO: 1,901,362) stands out as an obvious exception because it has more DEGs than other GO terms in the same category (sterol biosynthesis) (Figure 4 (C2)). As a result, GO: 1901362 had been divided into a different category (Figure 4 (C3)). Each category of the 13 DEGs contains two copies of the enzyme 3-hydroxy-3-methylglutaryl-CoA reductase (hmgcra), which is involved in the production of terpenoid backbones.

### 2.6. Gene Co-Expression Analysis Based on All Profiles

**Figure 5:**
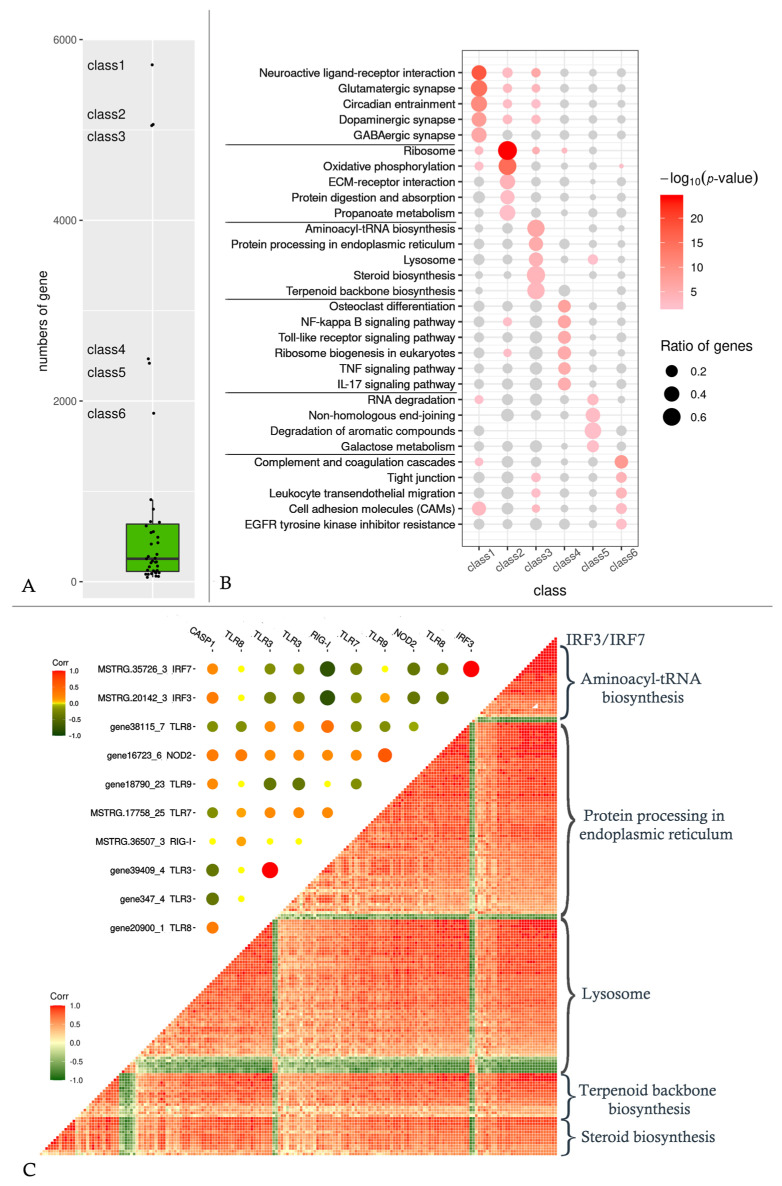
The curation of WGCNA analysis. (**A**) The scatter boxplot of gene counts for 39 classes. (**B**) The KEGG enrichment of the 6 outlier classes. The y-axis shows the top 5 enriched pathways of each class and the bubbles show the enrichment of the 6 classes on these selected pathways. (**C**) Upper triangle shows the correlation coefficients between IRF3/IRF7 and PRRs. The naming rule for the y-axis is GeneID_class NO. Symbol, and the tag of the x-axis is simplified to symbol. Lower triangle shows the correlation coefficients between IRF3/IRF7 and the genes in the top 5 enriched pathways of class 3.

After gene co-expression analysis, all genes were grouped into 39 classes (Table S2), with class 1 to class 6 having significantly more genes than the other classes (Figure 5A). The enrichment of each of these six gene classes was examined independently (Figure 5B).

The genes of class 1 are concentrated in neural signaling-related pathways, but the genes of these pathways are enriched in several classes, not just class 1. The ribosome and oxidative phosphorylation pathways are significantly enriched in class 2 genes.

Protein synthesis and sterol-related pathways are enriched in class 3 genes. The class 4 genes are clearly associated with cellular innate immunity, and they are enriched in signaling pathways such as NF-kappa B, toll-like receptor, and TNF, among others. Class 5 is thought to be associated with RNA degradation, while class 6 is found in the complement system and intercellular junctions.

It is worth noting that the two most important IFN regulators, IRF3 and IRF7, are both members of class 3. These two genes’ expression patterns did not correlate with nearly all PRRs (Figure 5C, upper triangle), and were even completely negative correlated with RIG-I. Despite this, these two genes were found to be positively associated with the majority of genes involved in aminoacyl–tRNA biosynthesis, protein processing in the endoplasmic reticulum (ER), lysosome, terpenoid backbone biosynthesis, and steroid biosynthesis (Figure 5C, lower triangle).

### 2.7. Validation of RNA-Seq Data with RT-qPCR

**Figure 6:**
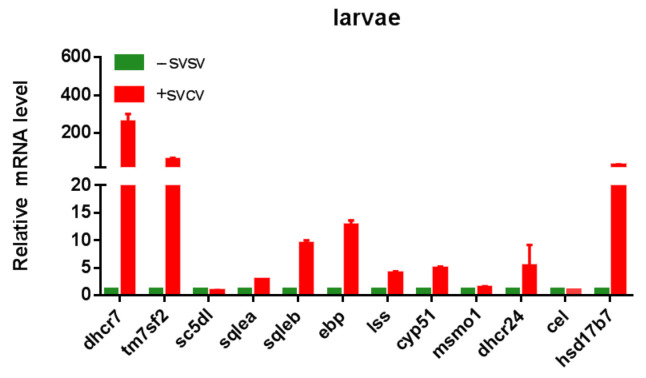
The RT-qPCR validation of DEGs of sterol biosynthesis.

We used RT-qPCR to test 12 genes on this pathway in light of the importance of the sterol biosynthesis pathway following viral infection in order to confirm the alterations regarding sterol biosynthesis genes of the zebrafish larvae. The RT-qPCR results were in line with the transcriptome, and in particular, dhcr7, hsd17b7, and tm7sf2 showed greater than 100-fold upregulation (Figure 6).

## 3. Discussion

Both previous studies [24,25,26,27,28] and our preliminary experiments showed that all four viruses can infect zebrafish. In our study, juvenile zebrafish were infected with these viruses and their expression profiles were analyzed at early stages of infection. The activation of type-I IFN pathway after infection in the results and the symptoms of the remaining zebrafish after sampling indicated that the infection experiments were successful. However, we compared our RNA-seq data to the genomes of all published strains of these four viruses, and no reads were mapped to any references. This may be related to the early sampling time when the viruses have not made enough copies to be detected.

We wanted to address two key questions through the data analysis. The first question was whether fish have a unified response to virus infection during the early stage and whether this response relies on the PRRs to switch on innate immunity. Second, do fish have different response systems to different viruses during the early phases of infection? Specifically, are there different responses to DNA and RNA viruses in fish?

Regardless of differences in the magnitude of gene expression change, the distribution of PBGs in all DEGs of the four viral infections (Figure 1B) demonstrates that different viral infections cause similar changes in fish. These changes involve a significant number of genes with highly consistent expression trends before and after virus infection. These genes function in the sterol-related pathways, protein metabolism, and innate immune system (Figure 1C), and the nervous system was enriched when virus-specific DEGs were also taken into account (Figure 3). Virus invasion in the infected fish we sampled was in its early stages. Surprisingly, no known PRR was upregulated, and nearly all were downregulated or unchanged. We suspect that at this point, the viruses had not yet been recognized by the host PRRs, and they were hosting the host cells in a frenzy to synthesize the proteins they required. We are unable to determine whether the elevated expression of genes involved in protein synthesis (Figure 3, group F) is due to viral hostage taking or the body’s antiviral reaction. Even though the majority of the genes in the “protein processing in endoplasmic reticulum” pathway were upregulated to reduce this stress, the abundance of newly generated proteins unavoidably stresses the host ER and causes the organism to go into stress mode.

The interaction of stress and the immune system in fish is obvious, as is the response of fish to various stressors. It is now widely accepted that chronic stressors suppress fish immune systems, both innate and acquired [12,13,14,15,16,17,18,19,20,21,22,23,24,25,26,27,28,29]. The neuroendocrine system most likely regulates this process via several hormones or other small molecules [11]. In response to chronic stimulation, for example, the body generally downregulates the inhibitory neurotransmitter γ-aminobutyric acid (GABA), which causes high cortisol levels and immunosuppression [30]. Our study’s systemic changes were highly consistent with fish responding to chronic stress. DEGs on neural signaling pathways, including GABA receptors, were mostly downregulated (Figure 3, group D). Furthermore, many important innate immune-related pathways were suppressed. For example, after four viruses’ infections, all DEGs in the arachidonic acid pathway were downregulated, and prostaglandin, an arachidonic acid metabolite, is strongly associated with immune activation [13]. Furthermore, large proportions of DEGs in complement and other cellular innate immune pathways were downregulated, including key genes in these pathways, such as C3 and RIG-I. (Figure 3, group B). In contrast, genes in the steroid biosynthesis pathway (Figure 3), which provides precursors for steroid hormones, were significantly upregulated. It is suggested that during the early stages of virus infection, the fish may be stressed, resulting in immunosuppression. When the expression profiles of SVCV and GCRV were compared, it was discovered that the high production of sterols and their derivatives likely rescued the suppressed immune system. According to the distribution of DEGs of both viruses in the three gene blocks of the nervous system (Figure 3, group D), sterol biosynthesis (Figure 3), and innate immune system (Figure 3, group B), SVCV downregulated neural signaling pathways, including GABA, while upregulating sterol biosynthesis-related genes, and also activated the initial immune response, including high expression of IRF3 and IRF7. GCRV, on the other hand, did not appear to be activated in either neural signaling pathways or sterol biosynthesis, and even showed a slightly opposite expression trend compared to the other viruses, despite having the most downregulated DEGs in its innate immune system.

It is hypothesized that stress mediated the high production of sterols and their derivatives as well as the occurrence of immunosuppression through the neuroendocrine system, but the former does not appear to be the cause of the latter; instead, the high production of sterols and derivatives may have rescued the suppressed immune system. Some immune activators are likely present in the high yield of sterols and their derivatives, in the same way that 7-DHC improves the effects of IFN production in mice [17,18,19,20,21,22,23,24,25,26,27,28,29,30,31].

The co-expression network analysis was used to classify all genes into 39 classes based on their expression patterns in order to further identify the pathways associated with IRF3 and IRF7, the key genes of the initial immune response. The first six classes contained significantly more genes than the other classes (Figure 5A), accounting for 70.54% of the total genes (22,700/32,177), with classes 1–3 containing more than 5000 genes per class and the remaining three classes containing around 2000 genes per class. The enrichment analysis revealed that each class’s function was distinct (Figure 5B). The upregulated PBGs were grouped into class 3, which is the focus of our study. While IRF3 and IRF7, two of the first key immune genes to be activated, were also members of class 3, these two genes were barely or negatively correlated with the PRRs (Figure 5C, upper triangle), and these PRRs were not clustered in class 4, the immune-related class (Figure 5C, upper triangle, Y-axis tags), implying that at the early infection stage, the PRRs in fish did not yet initiate the downstream immune response, but the organism had already begun to produce IFN to defend against the viruses. Additionally, IRF3 and IRF7, which belong to the same gene cluster, have a strong positive correlation with the majority of the genes in the top five enriched pathways of class 3. Two metabolic pathways, aminoacyl–tRNA biosynthesis and protein processing in ER, are involved in protein synthesis. In some studies, the defect of tRNA synthetase in zebrafish can cause excessive ER stress, which in turn leads to neuronal apoptosis [32]. The terpenoid backbone biosynthesis pathway is located upstream of steroid biosynthesis, and the lysosome pathway participates in the transmembrane transport of sterols [33,34]. In our study, this result demonstrates a positive correlation between protein, sterol, and IFN synthesis systems.

The results of differential expression, co-expression analysis, and RT-qPCR indicate that in the early stages of viral infection, fish have a universal response system that recognizes the virus before the PRRs. This system is most likely activated by the stress of ER overload, and it causes a surge in the production of sterols and their derivatives via the neuroendocrine system, ultimately increasing the synthesis of IFN to defend against virus invasion. Additionally, we think that this system does not distinguish between DNA and RNA viruses when it comes to recognition, and that flcn and trive2 (Figure 4A) are considered to be DEGs of DNA vs. RNA virus infections because the two DNA viruses are herpesviruses, which cause these two genes to express themselves in a manner that is more similar to that of controls and RNA viruses. The fact that there were so few DEGs between these two herpesviruses further demonstrates how similarly the fish responded to infection. Interestingly, despite the low number of DEGs, they were almost enriched in sterol synthesis-related GO terms, including the most highly upregulated gene, cyp1a, which is also a sterol-related gene [35], and the significantly differential expression of sterol-related genes in the organism responding to two RNA viruses previously discussed. All of these findings lead us to further hypothesize that variations in how this system responds to viral infections are most likely caused by the degree of activation of sterol-related pathways, and that this variability may be connected to the type of virus or the infectivity of the strain used in an experiment, though this is not yet proven.

## 4. Materials and Methods

### 4.1. Viral Infection and Fish

In this investigation, two virus challenge trials were carried out, with the testing samples being 3 dpf zebrafish larvae at 10 h after infection. For the former, the transcriptome sequencing was done with larvae infected by the four viruses CaHV, CyHV2, GCRV, and SVCV respectively and with physiological saline as control. Each group had three replicates and each replicate consisted of 40 larvae. As for the latter, samples for RT-qPCR were obtained via SVCV infection and physiological saline immersion of larvae. Each sample also contained 40 zebrafish larvae. The previously reported protocol was followed in all viral challenge trials [36].

### 4.2. RNA Extraction and Digital RNA Sequencing

Total RNA was extracted using RNAiso Plus (TaKaRa Bio, Beijing, China) according to the manufacturer’s protocol. The mRNA was enriched with Oligo(dT) magnetic beads and fragmented with fragmentation buffer after being assessed for purity and integrity with NanoDrop (Thermo Fisher Scientific, Wuhan, China) and Agilent 2100 (Agilent, Beijing, China).

The first strand cDNA was synthesized using random hexamers, and during the second strand cDNA synthesis, dTTP was replaced by dUTP to indicate strand specificity. The library was built after purification (AMPure XP beads), adaptor (including barcode) ligation, UDG degradation, and PCR amplification. The Agilent 2100 was used to inspect the quality of these libraries before they were used for paired-end sequencing on the HiSeqTM4000 (Illumina, Beijing, China). Under the accession number CRA004201, the clean data were uploaded to the GSA database: http://bigd.big.ac.cn/gsa (accessed on 18 February 2023) [37].

### 4.3. Data Processing and Basic Statistical Analysis

Using the default settings, NGSQCToolkit (v 2.3.3) [38] eliminated the adapters and the subpar data. To eliminate the potential bias introduced by PCR during the library construction process, the clean reads were clustered using barcodes by gencore (v 0.16.0) [39]. The reads were then mapped to the zebrafish genome (GRCz11) by HISAT2 (v 2.1.0) [40] and assembled by StringTie (v 1.3.5) [41]. Due to StringTie’s propensity for producing fusion genes during assembly, the fusion genes in the new GTF file were divided in accordance with the original zebrafish genome GFF file. Transcriptomic data were quantized by Salmon (v 1.3) [42]. The reference sequences used by Salmon consisted of all transcripts and decoy sequences, which are similar to transcripts in the zebrafish genome [43]. The index of reference sequences was built with the parameters “-k 23—keepDuplicates.” Salmon’s “quant” command with the parameters “—mimicBT2—useEM” was used to calculate the number of transcripts and genes. The non-coding transcripts were predicted using CPC2 (v 1.2.2) [44] and CPAT (v 0.1) together [45,46]. Genes lacking mRNA transcripts were excluded. By using BLAST and the nr database (e-value ≤ 1e-5), the novel ones in the remaining genes were annotated.

### 4.4. Identification of Differentially Expressed Genes (DEGs) and Analysis

The low expression genes with a total count of fewer than 10 in 15 samples were eliminated. The differential expression analysis of the remaining genes was performed using DESeq2 (v 1.24.0) [47]. DEGs were identified in this study using *p*-values (≤0.05) adjusted for false discovery rate (FDR) and fold changes (≥2) in expression level.

For GO enrichment analysis, TopGO [48] was used. The KEGG database was downloaded via its API interface, and the statistical test (Fisher’s exact test) for enrichment analysis was run using the R statistical language at the significance level of 0.05. The same genes were used in the co-expression analysis as in the differential expression analysis using the R package WGCNA (v 1.69) [49].

### 4.5. Validation of DEGs Using RT-qPCR

The Revert Aid First Strand cDNA Synthesis Kit (Thermo Fisher Scientific, Waltham, MA, USA) was used to synthesize cDNA, which were used as templates for RT-qPCR. The MonAmp SYBR Green qPCR Mix (high ROX) (Monas Bio., Shanghai, China) for RT-qPCR on the CFX Connect Real-Time PCR System (Bio-Rad Laboratories, Wuhan, China) was used. The fold change of the gene relative expression was obtained through the ΔΔCT treatment using the beta actin gene as calibrators. The primers for RT-qPCR are listed in Supplemental Table S3.

## Data Availability

The sample information and sequencing data have been submitted to the GSA database (http://bigd.big.ac.cn/gsa, accessed on 18 February 2023) with the accession number CRA004201.

## Supplementary Materials

The following supporting information can be downloaded at: https://www.mdpi.com/article/10.3390/ijms24054427/s1.
